# The Influence of Social Relationships on Third-Party Punishment: The Roles of Relationship Type Congruence and Threat Perception

**DOI:** 10.3390/bs16040482

**Published:** 2026-03-24

**Authors:** Zhijie Xiang, Yichen Zhu, Qinhan Zhang, Ersheng Chen, Xiaolu Wu

**Affiliations:** 1 School of Psychology, Zhejiang Normal University, Jinhua 321004, China; xzjxzj1@zjnu.edu.cn (Z.X.); qinhanzhang@zjnu.edu.cn (Q.Z.);; 2 School of Education, Zhejiang Normal University, Jinhua 321004, China

**Keywords:** social relationships, relationship type congruence, threat perception, third-party punishment, deterrence theory

## Abstract

Third-party punishment involves bystanders voluntarily incurring costs to punish norm violators, thereby maintaining social norms and cooperation. While prior research shows reduced punishment when the bystander and violator are friends, less is known about how the violator–victim relationship affects such punishment. Based on deterrence theory, punishment serves both to sanction violations and deter future threats. Accordingly, using the Dictator Game–third-party punishment paradigm across five experiments with a primary adult sample, this study investigated the impact of social relationships on third-party punishment, examined the mediating role of threat perception, and validated the applicability of deterrence theory within the context of third-party punishment. A pilot experiment confirmed that bystanders punish friends less than strangers. Experiment 1 showed that when the bystander and violator were friends, punishment was stronger if the violator and victim were also friends. Experiment 2 showed that congruent social relationships (e.g., all parties are friends) elicit greater punishment than incongruent ones. Experiment 3 demonstrated that threat perception mediates this effect: consistency increases threat perception, which in turn heightens punishment. In summary, consistency of social relationships increases third-party punishment, mediated by elevated threat perception. These findings support the use of deterrence theory in third-party punishment contexts and deepen our understanding of how social relationships shape punitive behavior.

## 1. Introduction

Third-party punishment (TPP) refers to the behavior of bystanders whose own interests are not directly harmed, yet who are willing to incur personal costs to punish violators (Fehr & Fischbacher, 2004). Its theoretical origins can be traced back to mechanisms for safeguarding public interests within socio-economic systems (Yamagishi, 1986), where group members spontaneously establish sanctions against violators, serving as a cornerstone for sustaining collective cooperation. However, early research had not yet explicitly proposed the term “third-party punishment”; instead, TPP was often observed as a driving factor behind other social phenomena (Fehr & Gächter, 2000; Hoffman et al., 1996). Building on a systematic integration of existing empirical evidence, Fehr and colleagues formally introduced and defined the concept of “third-party punishment” in 2004, establishing it as a core mechanism for maintaining social norms (Fehr & Fischbacher, 2004). Subsequently, researchers have systematically verified the existence and broad relevance of TPP through a series of empirical investigations. Foundational studies on TPP focused on adult populations, revealing its intrinsic role in sustaining norm enforcement. The research in this area was later extended to developmental stages, tracking the developmental trajectory of TPP in young children and eventually expanding into comparative studies with non-human primates, confirming the evolutionary uniqueness of TPP in humans (Fehr & Fischbacher, 2004; McAuliffe et al., 2015; Riedl et al., 2012). Furthermore, TPP plays an important role across multiple domains such as politics, economics, and culture. For instance, the public goods game paradigm based on TPP provides an operational theoretical framework for international climate cooperation, while in the economic domain, TPP has been shown to be a key mechanism for maintaining efficient cooperation in markets (S. Chen et al., 2021; Tavoni et al., 2011).

Having established the existence and functions of third-party punishment (TPP), research has shifted its focus to its antecedents, which are systematically organized along two main lines: “individual” and “contextual” factors. At the individual level, emotions such as anger and disgust significantly increase the willingness to punish, while moral outrage elevates both the intensity of this willingness and the likelihood of its enactment (Leliveld et al., 2012). Regarding personality traits, a high sense of social responsibility positively predicts the strength and persistence of punitive behavior, and high justice sensitivity directly reinforces the propensity to engage in TPP (Feng et al., 2016). Social status acts as a further moderating variable; individuals with higher status are more likely to take on the “enforcer” role and tend to invest disproportionate resources to amplify the impact of their punishment (Hu et al., 2016). Furthermore, TPP is influenced by age, with decision-making becoming increasingly systematic over the lifespan—older individuals tend to make punishment decisions in a more rational, rule-based manner, guided by fairness principles (Lee & Warneken, 2022). On the contextual level, TPP is modulated by multiple attributes of the violation itself. Within distributive frameworks, the degree of outcome inequity is positively correlated with the probability of punishment. The extent of deviation from a fair outcome (especially severe deviations) significantly increases the resources that are allocated to punishment. Additionally, attributions of the violator’s motive (e.g., self-interested motives) lead to a significant escalation in the severity of punishment (M. Chen et al., 2026; M. Chen, 2022). Empirical studies consistently emphasize the necessity of rigorously manipulating the social connections between the observer, violator, and victim within experimental paradigms. This underscores that beyond the individual- and context-oriented dimensions, social relationships constitute a crucial third dimension driving TPP, prompting a paradigm shift in research towards this social relational perspective (Baumgartner et al., 2012).

When third parties are required to administer punishment, they often struggle to remain objective. Specifically, their reactions to norm violations vary depending on the closeness of their social relationships with the violator and victim. Social relationships specifically refer to the psychological bonds that are formed between individuals during direct social interaction. From a functional perspective, they can be categorized into close and distant ties (Jia et al., 2012). Van Prooijen (2009) found that while a bystander’s initial reaction to a violation of social norms is typically to punish, their willingness to compensate increases significantly when the victim is a close friend. Xu et al. (2017) further demonstrated through experimental manipulation of social distance that bystanders punish strangers’ violations more severely than those of individuals who are close to them, although no significant difference was found in compensatory behavior. Campanha et al. (2011) provided complementary evidence from the perspective of distributive fairness, showing that bystanders are more likely to punish unfair allocation proposals made by strangers than identical proposals made by friends. The group identity of the violator also strongly influences third-party bystanders—they judge outgroup violators more harshly than ingroup violators for the same transgression and are more willing to protect ingroup victims (Graham et al., 1997; Sommers & Ellsworth, 2000). Researchers, drawing on ethnocentrism theory, note that individuals tend to exhibit altruistic behavior towards members of their own ethnicity, race, or other social groups, while showing indifference, distrust, or even hostility towards outgroup members. This systematic bias is identified as a cause for the differences observed in third-party punishment directed at violators from different groups (Brewer, 1999; Hewstone et al., 2002).

Do identity-based ingroup favoritism and outgroup discrimination directly drive third-party punishment behavior? A growing body of research using the third-party punishment game suggests that the cognitive mechanisms underlying TPP are more complex than simple ingroup bias(Jordan et al., 2016; Krasnow et al., 2016). Jordan et al. (2016) point out that when bystanders witness a norm violation, their punishment decision involves a complex cognitive evaluation mechanism. According to the Deterrence Hypothesis, bystanders use the observed behavior of the violator to predict their future behavioral patterns, assessing the potential risk of harm to themselves or their close associates (Krasnow et al., 2016). This prospective threat assessment directly drives an individual’s punitive decision; when the perceived potential threat is higher, individuals tend to impose harsher punishments as a means of preventing future harm. In this process, social relationships modulate punitive motivation by altering the cognitive judgment of “whether the violation poses a threat to oneself,” thereby affecting the perceived sense of threat. For instance, if an outgroup dictator harms an ingroup recipient, the punisher is more likely to infer “I could be next,” experiencing a heightened sense of threat and a stronger tendency to impose greater punishment. Conversely, if the perpetrator is also an ingroup member, their harm to an outgroup member does not reliably predict how they might treat the self, leading to a reduced sense of threat and weakened punitive motivation. Therefore, within the cognitive evaluation underlying third-party punishment, relationship type congruence—the alignment between the bystander–violator relationship and the violator–victim relationship—constitutes a core predictive factor. Bystanders assess relationship type congruence to calculate the probability of risk that the violation poses to themselves, perceive varying levels of threat, and consequently implement differentiated third-party punishment.

Existing research has confirmed that third-party punishment is influenced by social ties: individuals tend to favor those with close social bonds and reduce the intensity of punishment toward them (Qu et al., 2025; Xu et al., 2017; Zheng et al., 2024). However, current studies have mostly focused on the simple effects of the closeness and distance of social ties, without thoroughly exploring the internal cognitive mechanisms underlying third-party punishment decisions, and have neglected the complex processes by which individuals evaluate social ties, thus leaving a research gap. On this basis, the primary aim of the present study is to clarify the impact of relationship type congruence on third-party punishment and reveal the complex cognitive evaluation mechanism behind it—i.e., that individuals’ punitive behaviors are not merely expressions of favoritism, but selective decisions based on the relationship type congruence. The study proposes two core research questions: (1) Does relationship type congruence (i.e., whether the relationship between the transgressor and bystander is congruent with that between the transgressor and victim) affect the intensity of third-party punishment? (2) What is the psychological mechanism underlying third-party punishment, namely, does perceived threat play a mediating role between social ties and third-party punishment? By focusing on this perspective, this study aims to fill the gap in existing research, establish a clear position in the literature, and refine relevant theoretical models. To examine these questions, we recruited a sample of university students (aged 18–25 years). This population is commonly studied in research on social decision-making and provides a homogeneous sample that helps minimize potential confounds related to age and social experience. However, the use of this convenience sample limits the generalizability of our findings to other age groups, a point we address in the Section 4.2. Based on the above, in this study, we take college students as our research participants and employ the Dictator Game–third-party punishment paradigm (W. Chen et al., 2019; Raihani & Bshary, 2015). Four experiments were designed to investigate the influence of social relationship type congruence on third-party punishment and its underlying psychological mechanisms. Through a review of the relevant literature, this study proposes the following four research hypotheses:

**Hypothesis** **1.** *Different social relationships influence third-party punishment. Compared with when the violator is a stranger, third-party punishment decreases when the violator is a friend of the bystander.*

This hypothesis directly aligns with the extensive empirical evidence for ingroup favoritism. For instance, in their study on third-party punishment, Xu et al. (2017) found that individuals are more inclined to show favoritism towards ingroup members. We anticipate that this effect will be replicated and validated within the third-party punishment paradigm of the present study.

**Hypothesis** **2.** *Different social relationships influence third-party punishment. In situations where the violator and bystander are friends, third-party punishment increases when the violator and victim are also friends, compared with when the victim is a stranger.*

Although H1 predicts a general leniency towards friends, the situation becomes more complex when a transgression occurs between two friends. According to deterrence theory, individuals engage in third-party punishment to deter potential future mistreatment of themselves (Krasnow et al., 2016). We infer that in the scenario of “a friend harming a friend,” the bystander may consequently adopt a more severe punitive stance.

**Hypothesis** **3.** *The relationship type congruence influences third-party punishment. Compared with incongruent types of social relationships, third-party punishment increases when the relationship type between the violator and bystander is congruent with that between the violator and victim.*

This is based on the core premise of deterrence theory: punishment aims to prevent oneself from becoming a future victim (Jordan et al., 2016). When relationships are congruent, the bystander and victim occupy an equivalent position of potential harm, resulting in the highest perceived personal risk and thus the strongest punitive motivation. Incongruent relationships lead to lower personal risk and punitive motivation.

**Hypothesis** **4.** *Threat perception mediates the influence of the relationship type congruence on third-party punishment.*

Congruent social relationships elicit higher threat perception, leading individuals to enact more third-party punishment. Deterrence theory posits that punishment is driven by the assessment of future risk (Krasnow et al., 2016), while relationship type congruence serves as a key cognitive cue that directly elevates an individual’s perception of the threat of “becoming the next victim,” which constitutes the proximal psychological mechanism driving punitive decisions.

## 2. Method

### 2.1. Participants

A total of 512 undergraduate students participated in four experiments (pilot: 136; Experiment 1: 132; Experiment 2: 132; Experiment 4: 112). Participants were aged 18–25 years, had no history of mental illness or cognitive impairment, and had not participated in similar experiments. All participants provided written informed consent and received 50 RMB or course credit upon completion. Demographic characteristics (age, gender, academic major) for each experiment are detailed in Supplementary Materials Table S1.

### 2.2. Procedure

All four experiments were conducted in a quiet laboratory setting. Upon arrival, participants completed a brief demographic questionnaire and were presented with scenario materials that manipulated social relationships (friend vs. stranger). After a manipulation check (e.g., “hat is your relationship with the transgressor?”), participants rated perceived threat and made third-party punishment decisions (see Section 2.4). At the end of the session, participants were debriefed, compensated, and dismissed. All experimenters followed standardized scripts to ensure consistency

### 2.3. Statistical Analysis

Data were analyzed using IBM SPSS Statistics 26.0. Independent-samples *t*-tests and analyses of variance (ANOVAs) were used to examine the effects of social relationship, relationship type congruence, and unfairness level on third-party punishment intensity and perceived threat. Mediation analyses were conducted using Hayes’ PROCESS macro (Model 4) with 5000 bootstrap samples to test whether perceived threat mediated the relationship between relationship type congruence and punishment. Statistical assumptions (e.g., normality) were checked and met; detailed results of Shapiro–Wilk tests and procedures for outlier detection and missing data imputation are available in Supplementary Materials Table S2 and Text S1. The significance threshold was set at *α* = 0.05.

### 2.4. Experimental Paradigms and Materials

#### 2.4.1. Third-Party Punishment Game

A modified version of the Dictator Game, commonly referred to as the third-party punishment game (Jordan et al., 2016; Krasnow et al., 2016), was employed. In each trial, a transgressor (allocator) divided 100 RMB between themselves and a victim. Participants, acting as third-party bystanders, were endowed with 50 RMB and could spend a portion of this amount to punish the transgressor. The punishment cost was 1 RMB from the bystander for every 2 RMB deducted from the transgressor’s payoff. Participants were informed that their remaining endowment would be included in their participation fee (Fehr & Fischbacher, 2004).

#### 2.4.2. Scenario Manipulations

Social relationships were manipulated by asking participants to imagine that the transgressor was either a friend or a stranger. Allocation fairness was manipulated by varying the distribution ratio: 9:1 (extremely unfair), 6:4 (moderately unfair), or 5:5 (fair baseline). The full text of the scenario materials is provided in Supplementary Materials Text S2. Manipulation checks confirmed that participants perceived these scenarios as intended (see Results).

#### 2.4.3. Punishment Measurement

After viewing each allocation, participants answered two questions: (1) “Do you want to spend any of your 50 RMB to punish the transgressor?” (yes/no); (2) If yes, “How much do you want to spend?” (0–50 RMB). A comprehension check question was administered prior to the task; participants who answered incorrectly were excluded (exclusion details are reported in Results and summarized in Supplementary Materials Table S1).

## 3. Experimental Research

### 3.1. Pilot Experiment: The Effect of Social Relationship Between Transgressor and Bystander on Third-Party Punishment

#### 3.1.1. Experimental Purpose

The pilot experiment aimed to examine the effects of allocation fairness and the social relationship between the individual (i.e., the bystander) and the transgressor on third-party punishment, as well as to verify Hypothesis H1.

#### 3.1.2. Experimental Design

A power analysis using G*Power version 3.1.9.2 (α = 0.05; effect size f = 0.25; power = 0.80) indicated a required sample size of 98 participants. A total of 150 undergraduate students were initially recruited. After excluding 14 participants who failed the manipulation check or met other exclusion criteria, the final sample consisted of 136 participants (68 in the friend condition, 68 in the stranger condition). Demographic characteristics are summarized in Supplementary Table S1, and exclusion details are provided in Supplementary Table S3.

#### 3.1.3. Experimental Method

##### Participants

In accordance with previous relevant studies (S. Chen et al., 2021), a medium effect size (f = 0.25) was required. A power analysis using G*Power (α = 0.05; effect size = 0.25; power = 0.80) indicated a required sample size of 98 participants. To improve the statistical power, a total of 136 undergraduate students not majoring in psychology were recruited in this experiment, comprising 75 males and 61 females, with a mean age of 20.33 years (SD = 1.10). There were 68 participants in the friend condition group and 68 in the stranger condition group.

##### Manipulation of Social Relationship

Group allocation: Participants were randomly assigned to two groups. Only the social relationship between the transgressor and bystander (participant) was manipulated; the other two relationships (bystander–victim and transgressor–victim) were set as control variables and uniformly defined as stranger relationships to avoid confounding the core manipulation. The two groups were specified as follows: Group 1 (bystander–transgressor = friends) and Group 2 (bystander–transgressor = strangers).

Specific manipulation procedures: (1) Friend condition (Group 1): Participants were instructed to identify one close friend in their mind and write down his/her name (or nickname) and age; they were clearly informed that “this friend is the transgressor in the experimental scenario”, in order to strengthen the participants’ perception of being friends with the transgressor. All other roles (i.e., the victim) were set as strangers. (2) Stranger condition (Group 2): Participants were informed that “the transgressor in the experimental scenario is an unknown individual”, with no additional manipulation. (3) Manipulation reinforcement: After participants completed the imagination and writing task, the experimenter read standardized instructions to reinforce their perception of the relationship type (e.g., “Please keep the assigned relationship in mind; your subsequent decisions should be based on this relational scenario”), ensuring that the manipulation was congruent with the experimental design.

Manipulation check: Immediately after the manipulation, participants completed the Inclusion of Other in the Self (IOS) scale (only for the relationship between themselves and the transgressor) and answered manipulation check questions (e.g., “What relationship do you think you have with the transgressor?”). Participants who gave incorrect answers were reminded to reconfirm; data from those who still responded incorrectly were excluded to ensure the effectiveness of the core relationship manipulation.

#### 3.1.4. Experimental Procedure

First, instructions were provided. The experimenter informed participants that they were taking part in an experiment based on an imagined economic game scenario, where they needed to imagine the choices that they would make in specific allocation situations. Participants also read and were required to correctly understand the allocation rules. To avoid psychological influences from certain terms, roles were labeled as A/B/C in the experiment. The participant was always in the role of the bystander (C), while the violator was labeled A, and the victim was labeled B. Participants were told that A and B formed a temporary partnership, cooperated on a task, and received a 100 yuan reward, which was randomly deposited into A’s account. A decided the distribution, and B could not refuse.

During the formal experimental phase, social relationship manipulation was conducted first. Participants in the “friend” condition were instructed as follows: “Imagine that A is someone you know very well, and you have a good relationship. Please identify a specific person in your mind and write down their name (nickname), age, and gender.” Participants in the “stranger” condition received no such instruction.

**Figure 1:**
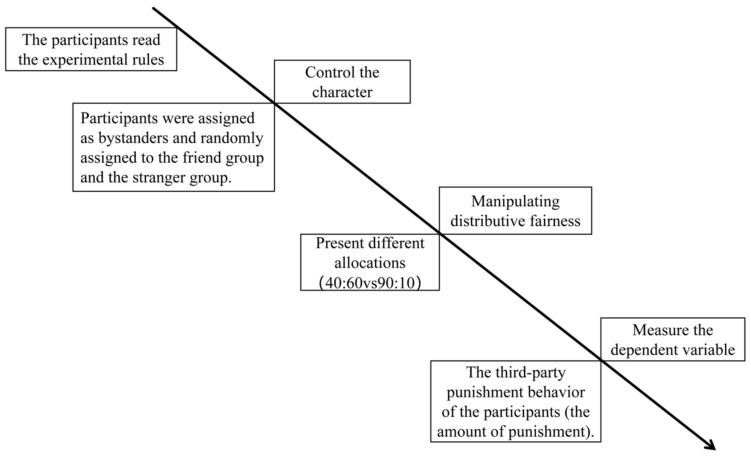
Flowchart of pilot study.

Subsequently, participants in both groups were randomly presented with the two unfair allocation scenarios (extremely unfair and slightly unfair) and made their third-party punishment decisions for each. The specific procedure is illustrated in Figure 1.

#### 3.1.5. Results of the Pilot Experiment

##### Manipulation Check of Social Relationship

An independent-samples *t*-test was conducted, with the score on the Inclusion of Other in the Self (IOS) scale as the dependent variable and the social relationship between the violator (A) and bystander (C) as the independent variable. The results revealed that the score under the friend condition was significantly higher than that under the stranger condition (M_Friend_ = 5.53, SD_Friend_ = 1.10; M_Stranger_ = 1.44, SD_Stranger_ = 0.58; *t*(136) = 27.097, *p* < 0.001), indicating that the manipulation of social relationship was effective.

##### Manipulation Check for Allocation Fairness

An independent-samples *t*-test was conducted, with participants’ perceived fairness rating as the dependent variable and the allocation fairness (slightly unfair vs. extremely unfair) as the independent variable. The results showed that the score under the slightly unfair condition was significantly higher than that under the extremely unfair condition (M_Slightly Unfair_ = 4.17, SD_Slightly Unfair_ = 1.49; M_Extremely Unfair_ = 1.90, SD_Extremely Unfair_ = 1.21; *t*(136) = 13.83, *p* < 0.001), indicating that the manipulation of allocation fairness was effective.

##### Measurement of Third-Party Punishment

**Figure 2:**
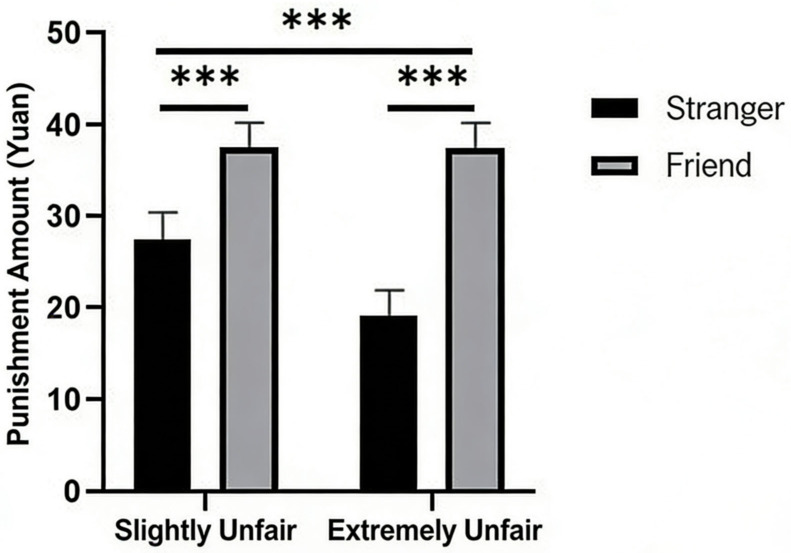
The effects of allocation fairness and social relationship on third-party punishment. Note. *** *p* < 0.001.

Using the level of third-party punishment as the dependent variable, the results showed that the main effect of allocation fairness was significant (M_Slightly Unfair_ = 6.49, SD_Slightly Unfair_ = 0.53; M_Extremely Unfair_ = 26.10, SD_Extremely Unfair_ = 1.21; F = 429.66, *p* < 0.001, *η_p_*^2^ = 0.76). The main effect of social relationship was also significant (F = 61.63; *p* < 0.001; *η_p_*^2^ = 0.32). Furthermore, the interaction between allocation fairness and social relationship was significant (F = 32.90; *p* < 0.001; *η_p_*^2^ = 0.20). A simple effect analysis of the interaction revealed that the simple effect of social relationship was significant under both the slightly unfair (F = 35.40; *p* < 0.001; *η_p_*^2^ = 0.21) and extremely unfair conditions (F = 57.29; *p* < 0.001; *η_p_*^2^ = 0.30). Similarly, the simple effect of allocation fairness was significant both under the stranger (F = 350.18; *p* < 0.001; *η_p_*^2^ = 0.72) and friend conditions (F = 112.39; *p* < 0.001; *η_p_*^2^ = 0.46). Statistical results are shown in Figure 2.

#### 3.1.6. Discussion of the Pilot Experiment

The pilot experiment manipulated social relationships through scenario imagination and measured participants’ third-party punishment using the Dictator Game–third-party punishment paradigm. Data analysis, with social relationship as the independent variable and the level of third-party punishment as the dependent variable, indicated that the social relationship significantly influenced third-party punishment. Specifically, compared with when the violator was a stranger, third-party punishment decreased when the violator was a friend of the bystander, supporting the experimental hypothesis.

This phenomenon can be explained by deterrence theory, according to which individuals engage in third-party punishment to prevent themselves from being treated poorly in the future (Krasnow et al., 2016). In our experiment, when the violator made an unfair allocation to a stranger, and the bystander was a friend of the violator, the bystander predicted a lower likelihood of being treated unfairly themselves, resulting in lower third-party punishment. Therefore, what happens when the violator makes an unfair allocation to their own friend? In this case, as a friend of the violator as well, does the bystander’s prediction of the likelihood of being treated unfairly change, and consequently, does their punitive behavior also change? Therefore, Experiment 1 was used to investigate the influence of the social relationship between the violator and victim on third-party punishment when the bystander and violator are friends.

### 3.2. Experiment 1: The Effect of the Social Relationship Between Transgressor and Victim on Third-Party Punishment

#### 3.2.1. Experimental Purpose

Experiment 1 was intended to examine the influence of the social relationship between the transgressor and victim on individuals’ third-party punishment when the transgressor and bystander were friends, in order to verify Hypothesis H2.

#### 3.2.2. Experimental Design

A two-factor mixed design was adopted in this experiment. The first independent variable was the social relationship between the transgressor and bystander, with two levels being implemented: the friend condition and the stranger condition (between-subjects). The second independent variable was allocation fairness, with two levels: the moderately unfair condition and the extremely unfair condition (within-subjects). The dependent variable was participants’ third-party punishment behavior.

#### 3.2.3. Experimental Method

##### Participants

A power analysis using G*Power (α = 0.05; effect size f = 0.25; power = 0.80) indicated a required sample size of 98 participants. A total of 150 undergraduate students were initially recruited. After excluding 18 participants who failed the manipulation check or met other exclusion criteria, the final sample consisted of 132 participants (66 in the transgressor–victim friend condition, 66 in the transgressor–victim stranger condition). Demographic characteristics are summarized in Supplementary Table S1, and exclusion details are provided in Supplementary Table S3.

##### Manipulation of Social Relationship

Group allocation: Participants were randomly assigned to two groups. The relationship between the bystander (participant) and transgressor (A) was fixed as friendship in both groups, and only the social relationship between the transgressor (A) and victim (B) differed. Irrelevant variables (e.g., experimental scenario, instructions) were kept consistent to minimize experimental confounding. The two groups were designed as follows: Group 1 (transgressor–victim = friends) and Group 2 (transgressor–victim = strangers).

Specific manipulation procedures: (1) Relationship priming: All participants first read a standardized introductory statement clarifying that “the transgressor in the experimental scenario is your friend A”, in order to strengthen participants’ perception of the friendship between themselves and the transgressor (A), laying a foundation for the experimental manipulation and ensuring that all participants completed the experiment under the same bystander–transgressor relationship premise. (2) Group-specific reading materials: Participants read corresponding experimental materials according to their assigned group, and the relationship between the transgressor and victim was manipulated through scenario descriptions. The materials were in strict accordance with the following settings: For the friend condition (Group 1), the reading material stated that “Your friend A and his/her friend B have agreed to form a partnership to jointly complete a remunerative task. After the task, the reward will be uniformly issued to your friend A’s account, and A will be responsible for distributing the reward between the two.” For the stranger condition (Group 2), the reading material stated that “Your friend A and a randomly selected individual B form a temporary partnership to jointly complete a remunerative task. The two have had no interaction or emotional bond before. After the task, the reward will be uniformly issued to your friend A’s account, and A will be responsible for distributing the reward between the two.” (3) Reading requirements: Participants were instructed to read the materials carefully and keep the relationship type between the transgressor (A) and victim (B) in the scenario in mind. Subsequent third-party punishment decisions were required to be made based on this scenario to avoid invalid results caused by inadequate comprehension of the materials.

Manipulation check: Immediately after reading the materials, participants completed a manipulation check: “Please identify the relationship type between Transgressor A and Victim B in the experimental scenario. (Options: A. Friend B. Stranger)”. Participants responded on the spot. Data from participants whose answers were incongruent with the actual transgressor–victim relationship in their group (i.e., incorrect responses) were excluded to ensure that the core relationship manipulation achieved the expected effect. Participants with correct responses proceeded to the subsequent experimental tasks.

#### 3.2.4. Experimental Procedure

First, instructions were provided. The experimenter informed participants that they were taking part in an experiment based on an imagined economic game scenario, where they needed to imagine the choices that they would make in specific allocation situations. Participants also read and were required to correctly understand the allocation rules. To avoid psychological influences from certain terms, roles were labeled A, B, and C. The participant was always in the role of the bystander (C), while the violator was labeled A, and the victim was labeled B. All participants were told that “A is someone you know very well, and you have a good relationship. Please identify a specific person in your mind and write down their name (nickname), age, and gender.”

During the formal experiment phase, social relationship manipulation was conducted first. Participants in the “friend” condition group read the following: “Your friend A and his/her friend B agreed to form a partnership …” Participants in the “stranger” condition group read the following: “Your friend A and a randomly assigned person B temporarily formed a partnership …”

**Figure 3:**
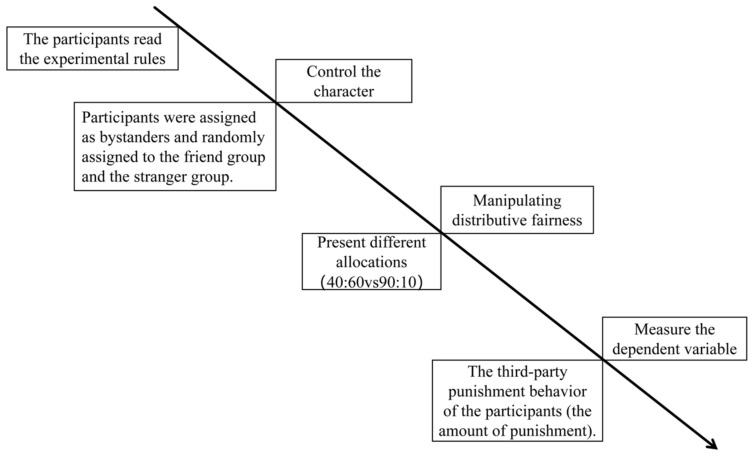
Flowchart of Experiment 1.

Subsequently, participants in both groups were randomly presented with the two unfair allocation scenarios (slightly unfair and extremely unfair) and made their third-party punishment decisions for each. Following this, they rated the fairness of A’s allocation, which served as the manipulation check for the independent variable of “allocation fairness.” The specific procedure is illustrated in Figure 3.

#### 3.2.5. Results of Experiment 1

##### Manipulation Check for Allocation Fairness

An independent-samples *t*-test was conducted, using participants’ perceived fairness ratings as the dependent variable and the allocation fairness (slightly unfair vs. extremely unfair) as the independent variable. The results revealed that the score under the slightly unfair condition was significantly higher than under the extremely unfair condition (M_Slightly unfair_ = 3.82, SD_Slightly unfair_ = 1.73; M_Extremely unfair_ = 2.15, SD_Extremely unfair_ = 1.66; *t*(131) = 8.01, *p* < 0.001), indicating that the manipulation of allocation fairness was effective.

##### Measurement of Third-Party Punishment

Using the level of third-party punishment as the dependent variable, the results showed a significant main effect of allocation fairness (M_Slightly unfair_ = 5.65, SD_Slightly unfair_ = 5.70; M_Extremely unfair_ = 26.89, SD_Extremely unfair_ = 15.58; F = 483.09, *p* < 0.001, *η_p_*^2^ = 0.79). The main effect of social relationship was also significant (F = 82.43; *p* < 0.001; *η_p_*^2^ = 0.39). Furthermore, the interaction between allocation fairness and social relationship was significant (F = 47.15; *p* < 0.001; *η_p_*^2^ = 0.27).

**Figure 4:**
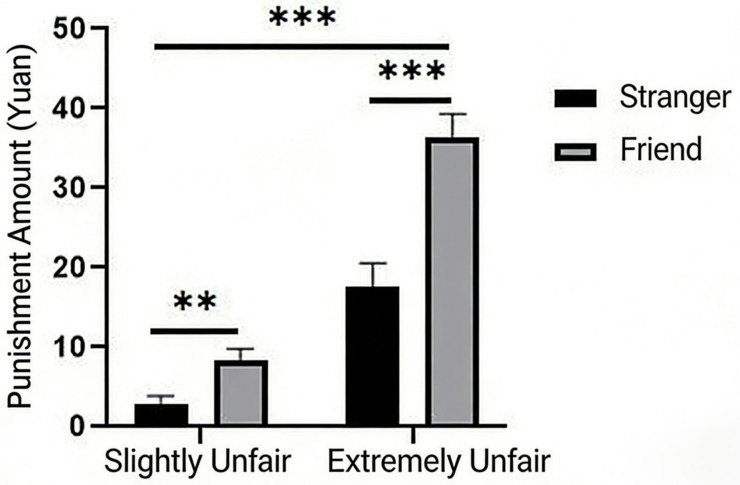
The effects of distributive fairness and social relationships on third-party punishment. Note. ** *p* < 0.0, *** *p* < 0.001.

A simple effect analysis of the interaction showed that the simple effect of social relationship was significant under both the slightly unfair (F = 40.07; *p* < 0.001; *η_p_*^2^ = 0.24) and extremely unfair conditions (F = 75.23; *p* < 0.001; *η_p_*^2^ = 0.38). Similarly, the simple effect of allocation fairness was significant both in the scenario where the violator and victim were strangers (F = 114.20; *p* < 0.001; *η_p_*^2^ = 0.47) and when they were friends (F = 416.04; *p* < 0.001; *η_p_*^2^ = 0.76). Statistical results are shown in Figure 4.

#### 3.2.6. Discussion of Experiment 1

Experiment 1 was conducted to examine the effect of the social relationship between the violator and victim on third-party punishment, and the results supported the experimental hypothesis. Specifically, under the condition that the violator and bystander were friends, compared with when the violator and victim were strangers, individuals exhibited increased third-party punishment behavior.

This finding suggests that the relationship type congruence may play a key role. When the violator and bystander are friends, and the violator and victim are also friends, the relationship types are congruent; when the violator and victim are strangers, the relationship types are incongruent. The observed pattern indicates that congruent relationship types may heighten bystanders’ anticipation of potentially experiencing similar unfair treatment themselves, thereby increasing third-party punishment. Experiment 2 was designed to directly investigate the effect of relationship type congruence on third-party punishment.

### 3.3. Experiment 2: The Effect of Relationship Type Congruence Between Transgressor–Bystander and Transgressor–Victim on Third-Party Punishment

#### 3.3.1. Experimental Purpose

Experiment 2 aims to investigate the effect of the congruence between the social relationship of the violator and the bystander and that of the violator and the victim on third-party punishment, thereby testing Hypothesis H3.

#### 3.3.2. Experimental Design

A two-factor mixed design was adopted in this experiment. The first independent variable was the relationship type congruence, with two levels: congruent social relationships and incongruent social relationships (between-subjects). The second independent variable was allocation fairness, with two levels: the moderately unfair condition and the extremely unfair condition (within-subjects). The dependent variable was participants’ third-party punishment behavior.

#### 3.3.3. Experimental Method

##### Participants

A power analysis using G*Power (α = 0.05; effect size f = 0.25; power = 0.80) indicated a required sample size of 98 participants. A total of 150 undergraduate students were initially recruited. After excluding 30 participants who failed the manipulation check or met other exclusion criteria, the final sample consisted of 120 participants (60 in the congruent condition, 60 in the incongruent condition). Demographic characteristics are summarized in Supplementary Table S1, and exclusion details are provided in Supplementary Table S3.

##### Manipulation of Social Relationship

**Group allocation:**

Participants were randomly assigned to two major groups (congruent and incongruent conditions), with four subgroups in total. Irrelevant variables (e.g., experimental scenario, instructions, task settings) were kept consistent to minimize experimental confounding. The specific design of each group followed the logic of relationship type congruence strictly:

(1) Congruent condition (two subgroups): The social relationship type between the transgressor (A) and bystander (C, participant) was fully congruent with that between the transgressor (A) and victim (B).

① Congruent Subgroup 1: Transgressor (A) and bystander (C, participant) were friends; transgressor (A) and victim (B) were friends.

② Congruent Subgroup 2: Transgressor (A) and bystander (C, participant) were strangers; transgressor (A) and victim (B) were strangers.

(2) Incongruent condition (two subgroups): The social relationship type between the transgressor (A) and bystander (C, participant) was incongruent with that between the transgressor (A) and victim (B), covering two different scenarios.

① Incongruent Subgroup 1: Transgressor (A) and bystander (C, participant) were friends; transgressor (A) and victim (B) were strangers.

② Incongruent Subgroup 2: Transgressor (A) and bystander (C, participant) were strangers; transgressor (A) and victim (B) were friends.

**Specific manipulation procedures:**

(1) Group priming: All participants first read a standardized introductory statement, which clarified that the core purpose of the experiment was to explore “the association between social relationships and decision-making behavior”. According to their randomly assigned subgroups, participants then received corresponding relationship manipulation instructions to confirm the relationships between themselves (bystander, C), the transgressor (A), and the victim (B).

(2) Subgroup-specific reading materials: Participants read corresponding experimental materials based on their subgroups, and the social relationships among the three roles were manipulated through scenario descriptions. The material content was designed in line with the research logic of Lieberman and Linke (2007), with specific settings as follows:

① Congruent Subgroup 1: The reading material stated that “Your friend A and his/her friend B have agreed to form a partnership to jointly complete a remunerative task. After the task, the reward will be uniformly issued to your friend A’s account, and A will be responsible for distributing the reward between the two. As a bystander, you witness the entire task process and reward allocation between the two.”

② Congruent Subgroup 2: The reading material stated that “A randomly selected individual A and a randomly selected individual B form a temporary partnership with no prior interaction or emotional bond. They jointly complete a remunerative task. After the task, the reward will be uniformly issued to A’s account, and A will be responsible for distributing the reward between the two. As a bystander, you are a stranger to both A and B, and you witness the entire task process and reward allocation between the two.”

③ Incongruent Subgroup 1: The reading material stated that “Your friend A and a randomly selected individual B form a temporary partnership with no prior interaction or emotional bond. They jointly complete a remunerative task. After the task, the reward will be uniformly issued to your friend A’s account, and A will be responsible for distributing the reward between the two. As a bystander, you witness the entire task process and reward allocation between the two.”

④ Incongruent Subgroup 2: The reading material stated that “A randomly selected individual A and his/her friend B have agreed to form a partnership to jointly complete a remunerative task. After the task, the reward will be uniformly issued to A’s account, and A will be responsible for distributing the reward between the two. As a bystander, you are a stranger to A, and you witness the entire task process and reward allocation between the two.”

(3) Reading requirements: Participants were instructed to read the materials carefully, keeping the relationship types between themselves and A and between A and B, as well as the consistency of such relationships, in mind. Subsequent third-party punishment decisions were required to be made based on this scenario to avoid invalid results caused by inadequate comprehension of the materials.

**Manipulation check:**

Immediately after reading the materials, participants completed a manipulation check with a single item, designed strictly in accordance with experimental requirements, as follows: “To what extent do you think you are similar to Victim B?” A 7-point rating scale was adopted (1 = not at all similar; 7 = extremely similar), and participants responded based on their actual perceptions. Meanwhile, supplementary confirmation of relationship cognition was conducted (through brief inquiries after rating, without separate items) to ensure that participants clearly understood the relationship types between themselves and A and between A and B, and their consistency. Data from participants with relationship cognition biases were excluded to ensure that the core manipulation of relationship type congruence achieved the expected effect. Participants with valid responses proceeded to the subsequent experimental tasks.

#### 3.3.4. Experimental Procedure

First, instructions were provided. The experimenter informed participants that they were taking part in experiment based on an imagined economic game scenario, where they needed to imagine the choices that they would make in specific allocation situations. Participants also read and were required to correctly understand the allocation rules. To avoid psychological influences from certain terms, roles were labeled A, B, and C. The participant was always in the role of the bystander (C), while the violator was labeled A, and the victim was labeled B.

Participants in the congruent CB friend group and the incongruent CB friend group were told that “A is someone you know very well, and you have a good relationship. Please identify a specific person in your mind and write down their name (nickname), age, and gender.” Participants in the congruent CB stranger group and the incongruent CB stranger group received no such instruction.

During the formal experiment phase, social relationship manipulation was conducted first. Participants assigned to the congruent CB friend group and the incongruent CB stranger group read the following: “Your friend A and his/her friend B agreed to form a partnership …” Participants assigned to the congruent CB stranger group and the incongruent CB friend group read the following: “Your friend A and a randomly assigned person B temporarily formed a partnership …”

**Figure 5:**
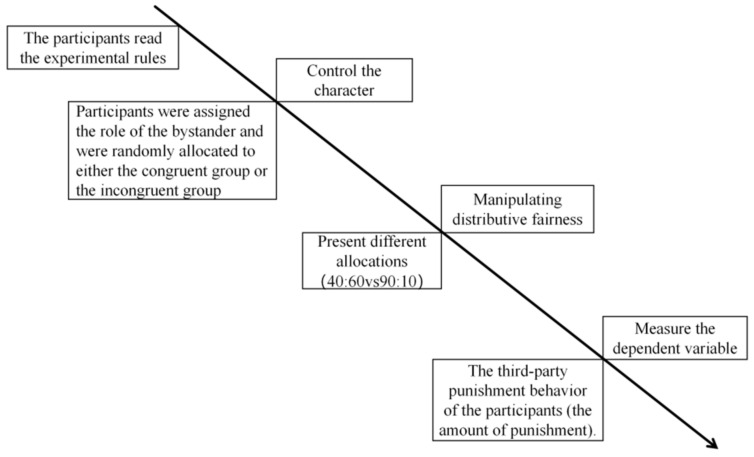
Flowchart of Experiment 2.

Subsequently, all participants were randomly presented with the two unfair allocation scenarios (slightly unfair and extremely unfair) and made their third-party punishment decisions for each. Following this, they rated the fairness of A’s allocation, which served as the manipulation check for the independent variable of “allocation fairness.” The specific procedure is illustrated in Figure 5.

#### 3.3.5. Results of Experiment 2

##### Manipulation Check for Social Relationship Type Congruence

An independent-samples *t*-test was conducted, with the score for the manipulation check question as the dependent variable and social relationship type congruence as the independent variable. The results revealed that the score under the congruent condition was significantly higher than under the incongruent condition (M _Congruent_ = 5.68, SD _Congruen_ = 0.73; M _Incongruent_ = 2.47, SD _Incongruent_ = 0.98; *t*(120) = 20.41, *p* < 0.01), indicating that the manipulation of social relationship type congruence was effective.

##### Manipulation Check for Allocation Fairness

An independent-samples *t*-test was conducted, with participants’ perceived fairness rating as the dependent variable and the allocation fairness (slightly unfair vs. extremely unfair) as the independent variable. The results showed that the score under the slightly unfair condition was significantly higher than under the extremely unfair condition (M_Slightly unfair_ = 4.45, SD_Slightly unfair_ = 1.56; M_Extremely unfair_ = 2.37, SD _Extremely unfair_ = 1.70; *t*(120) = 9.91, *p* < 0.001), indicating that the manipulation of allocation fairness was effective.

##### The Influence of Social Relationship Type Congruence on Third-Party Punishment

**Figure 6:**
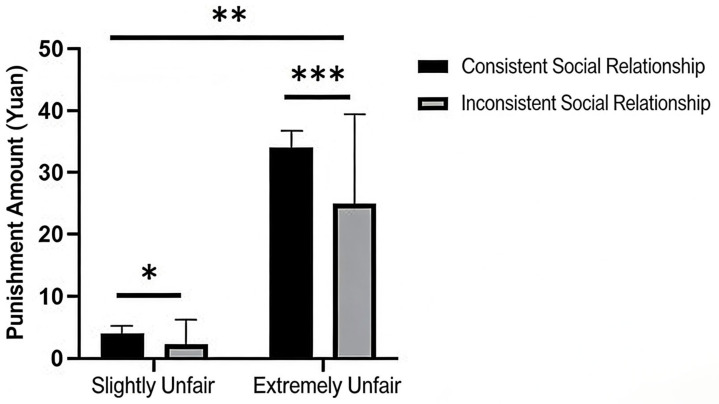
The impact of allocation fairness and relationship type congruence on third-party punishment. Note. * *p* < 0.05, ** *p* < 0.0, *** *p* < 0.001.

Using the level of third-party punishment as the dependent variable, the results showed a significant main effect of allocation fairness (M_Slightly unfair_ = 3.17, SD_Slightly unfair_ = 4.53; M_Extremely unfair_ = 29.60, SD_Extremely unfair_ = 13.30; F = 551.53, *p* < 0.001, *η_p_*^2^ = 0.82). The main effect of social relationship type congruence was also significant (F = 17.17; *p* < 0.001; *η_p_*^2^ = 0.13). Furthermore, the interaction between allocation fairness and social relationship type congruence was significant (F = 10.04; *p* < 0.01; *η_p_*^2^ = 0.08). A simple effect analysis of the interaction revealed that the simple effect of congruence was significant under both the slightly unfair (F = 5.07; *p* = 0.026; *η_p_*^2^ = 0.041) and extremely unfair conditions (F = 15.26; *p* < 0.001; *η_p_*^2^ = 0.115). Similarly, the simple effect of allocation fairness was significant both under the congruent (F = 355.21; *p* < 0.001; *η_p_*^2^ = 0.75) and incongruent conditions (F = 206.37; *p* < 0.001; *η_p_*^2^ = 0.64). Statistical results are shown in Figure 6.

#### 3.3.6. Discussion of Experiment 2

Experiment 2 was conducted to investigate the influence of social relationship type congruence on third-party punishment, and the results support the experimental hypothesis. Specifically, compared with incongruent social relationships, individuals’ third-party punishment increased when the social relationship type between the violator and victim was congruent with that between the violator and bystander (i.e., both were friends or both were strangers). Further separate ANOVA analyses under congruent and incongruent conditions revealed that when the violator and bystander were friends, third-party punishment decreased, although the difference was not significant. This pattern is consistent with findings from previous research and supports the hypothesis of ingroup favoritism theory (Krasnow et al., 2016).

According to deterrence theory, threat perception may play a crucial mediating role. Deterrence theory posits that bystanders infer the likelihood of themselves receiving unfair treatment based on the violator’s current behavior (Krasnow et al., 2016). It is plausible that when social relationship types are congruent, individuals perceive a higher level of threat, leading them to engage in more severe third-party punishment to deter the violator and prevent themselves from experiencing similar unfair treatment in the future. A review of the literature on threat perception also indicates that a high level of perceived threat motivates individuals to take proactive measures to protect themselves (Zou et al., 2025). Therefore, Experiment 3 was designed to investigate the mediating role of threat perception and address the question of why social relationship type congruence influences third-party punishment.

### 3.4. Experiment 3: The Mediating Role of Threat Perception in the Effect of Relationship Type Congruence Between Transgressor–Bystander and Transgressor–Victim on Third-Party Punishment

#### 3.4.1. Experimental Purpose

Experiment 3 aimed to explore the role of threat perception in the effect of relationship type congruence on third-party punishment, in order to verify Hypothesis H4.

#### 3.4.2. Experimental Design

A two-factor mixed design was adopted in this experiment. The first independent variable was the relationship type congruence, with two levels: congruent social relationships and incongruent social relationships (between-subjects). The second independent variable was allocation fairness, with two levels: the moderately unfair condition and the extremely unfair condition (within-subjects). The dependent variable was participants’ third-party punishment behavior.

#### 3.4.3. Experimental Method

##### Participants

A power analysis using G*Power (α = 0.05; effect size f = 0.25; power = 0.80) indicated a required sample size of 98 participants. A total of 150 undergraduate students were initially recruited. After excluding 38 participants who failed the manipulation check or met other exclusion criteria, the final sample consisted of 112 participants (56 in the congruent condition, 56 in the incongruent condition). Demographic characteristics are summarized in Supplementary Table S1, and exclusion details are provided in Supplementary Table S3.

##### Manipulation of Social Relationship

The manipulation procedure was identical to that used in Experiment 2.

#### 3.4.4. Experimental Procedure

First, instructions were provided. The experimenter informed participants that they were taking part in an experiment based on an imagined economic game scenario, where they needed to imagine the choices that they would make in specific allocation situations. Participants also read and were required to correctly understand the allocation rules. To avoid psychological influences from certain terms, roles were labeled A, B, and C. The participant was always in the role of the bystander (C), while the violator was labeled A, and the victim was labeled B.

Participants in the congruent BC friend group and the incongruent BC friend group were told that “A is someone you know very well, and you have a good relationship. Please identify a specific person in your mind and write down their name (nickname), age, and gender.” Participants in the congruent BC stranger group and the incongruent BC stranger group received no such instruction.

During the formal experiment phase, social relationship manipulation was conducted first. Participants assigned to the congruent BC friend group and the incongruent-BC stranger group read the following: “Your friend A and his/her friend B agreed to form a partnership …” Participants assigned to the congruent BC stranger group and the incongruent BC friend group read the following: “Your friend A and a randomly assigned person B temporarily formed a partnership …”

**Figure 7:**
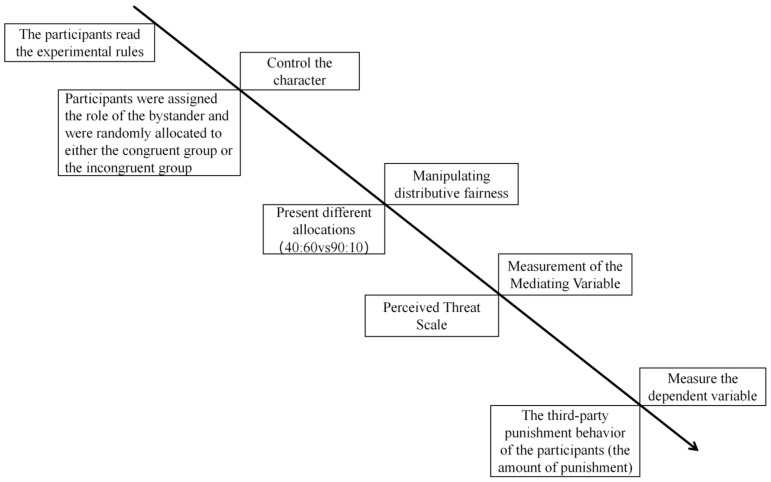
Flowchart of Experiment 3.

Subsequently, all participants were randomly presented with the two unfair allocation scenarios (slightly unfair and extremely unfair). Following the presentation of each scenario, they completed a threat perception scale to measure their level of perceived threat and then made their third-party punishment decision. Finally, they rated the fairness of A’s allocation, which served as the manipulation check for the independent variable of “allocation fairness.” The specific procedure is illustrated in Figure 7.

#### 3.4.5. Results

##### Manipulation Check for Social Relationship Type Congruence

The independent variable was relationship type congruence (dichotomous), and the dependent variable was the score of the manipulation check item (continuous). Given the normality of the data, the independent-samples *t*-test was appropriate for comparing the mean differences in the continuous dependent variable between the two groups and verifying the effectiveness of our manipulation. An independent-samples *t*-test was conducted, with the score for the manipulation check question as the dependent variable and social relationship type congruence as the independent variable. The results revealed that the score under the congruent condition was significantly higher than under the incongruent condition (M_Congruent_ = 5.56, SD_Congruent_ = 0.72; M_Incongruent_ = 2.54, SD_incongruent_ = 0.97; *t*(112) = 19.33, *p* < 0.01), indicating that the manipulation of social relationship type congruence was effective.

##### Manipulation Check for Allocation Fairness

The independent variable was allocation fairness (dichotomous), and the dependent variable was perceived fairness rating (continuous). The independent-samples *t*-test was used to compare the mean differences in the continuous dependent variable between the two groups and validate the effectiveness of our manipulation. An independent-samples *t*-test was conducted, with participants’ perceived fairness rating as the dependent variable and allocation fairness (slightly unfair vs. extremely unfair) as the independent variable. The results showed that the score under the slightly unfair condition was significantly higher than under the extremely unfair condition (M Slightly unfair = 4.25, SD Slightly unfair = 1.49; M Extremely unfair = 2.00, SD Extremely unfair = 1.76; *t*(112) = 10.36, *p* < 0.001), indicating that the manipulation of allocation fairness was effective.

##### The Influence of Social Relationship Type Congruence on Third-Party Punishment

The independent variables were the categorical variables, and the dependent variable was third-party punishment intensity (continuous). With normally distributed data, the adopted analytical method allowed for the examination of main and interaction effects to explore the relationships among variables. Using the level of third-party punishment as the dependent variable, the results showed a significant main effect of allocation fairness (M_Slightly unfair_ = 3.20, SD_Slightly unfair_ = 5.12; M_Extremely unfair_ = 28.24, SD_Extremely unfair_ = 13.97; F = 468.02, *p* < 0.01, *η_p_*^2^ = 0.81). The main effect of social relationship type congruence was also significant (F = 11.20; *p* < 0.01; *η_p_*^2^ = 0.09). Furthermore, the interaction between allocation fairness and social relationship type congruence was significant (F = 7.46; *p* < 0.01; *η_p_*^2^ = 0.06). A simple effect analysis of the interaction revealed that the simple effect of congruence was significant under both the slightly unfair (F = 4.13; *p* = 0.045; *η_p_*^2^ = 0.036) and extremely unfair conditions (F = 10.64; *p* = 0.001; *ηp*^2^ = 0.088). Similarly, the simple effect of allocation fairness was significant both under the congruent (F = 296.83; *p* < 0.001; *η_p_*^2^ = 0.73) and incongruent conditions (F = 178.65; *p* < 0.001; *η_p_*^2^ = 0.62).

##### Further ANOVA Analysis Under Conditions of Social Relationship Type Congruence or Incongruence

(1) The Influence of Social Relationship Type on Third-Party Punishment Under Congruent Conditions

Using the level of third-party punishment as the dependent variable, the results showed a significant main effect of allocation fairness (M_Slightly unfair_ = 4.17, SD = 5.88; M_Extremely unfair_ = 32.37, SD = 13.85; F = 290.74, *p* < 0.001, *η_p_*^2^ = 0.84). The main effect of social relationship type was not significant (F = 2.21; *p* > 0.05; *η_p_*^2^ = 0.04). Under the slightly unfair condition, when the violator–bystander and violator–victim relationships were congruent (both stranger relationships), the level of third-party punishment was higher (M congruent BC stranger = 5.39, SD = 5.79; M congruent BC friend = 2.95, SD = 5.82). Similarly, under the extremely unfair condition, when both relationships were congruent stranger relationships, the level of third-party punishment was higher (M congruent BC stranger = 34.55, SD = 12.40; M congruent BC friend = 30.18, SD = 15.06).

(2) The Influence of Social Relationship Type on Third-Party Punishment Under Incongruent Conditions

**Figure 8:**
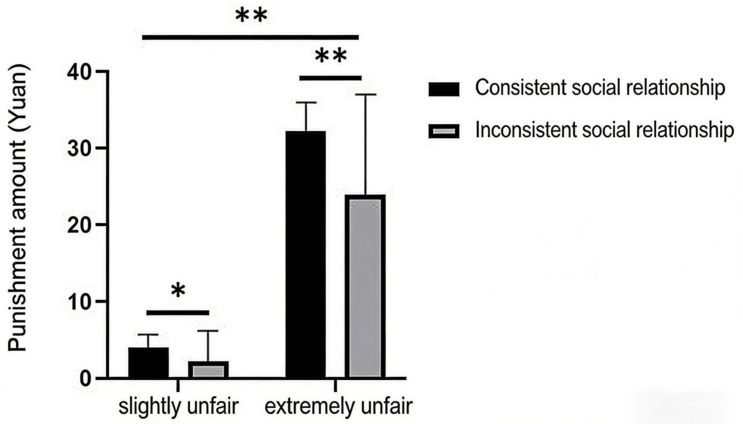
Effects of Distributive Fairness and Social Relationship Type Consistency on Third-Party Punishment. Note. * *p* < 0.05, ** *p* < 0.0.

Following the same statistical logic as described above, using the level of third-party punishment as the dependent variable, the results showed a significant main effect of allocation fairness (M_Slightly unfair_ = 2.23, SD = 4.04; M_Extremely unfair_ = 24.11, SD = 12.94; F = 177.03, *p* < 0.001, *η_p_*^2^ = 0.77). The main effect of social relationship type was not significant (F = 0.59; *p* > 0.05; *η_p_*^2^ = 0.01). Under the slightly unfair condition, when the social relationships were incongruent (violator–bystander are strangers, violator–victim are friends), the level of third-party punishment was higher (M _incongruent BC friend_ = 2.86, SD = 4.40; M _incongruent BC stranger_ = 1.61, SD = 3.61). Under the extremely unfair condition, a similar pattern was observed with incongruent relationships (violator–bystander are strangers, violator–victim are friends), resulting in higher punishment (M _incongruent BC friend_ = 25.00, SD = 13.40; M _incongruent BC stranger_ = 23.21, SD = 12.64). Statistical results are shown in Figure 8.

##### Mediation Effect Test for Threat Perception

To examine the mediating role of threat perception, a Bootstrap test was conducted based on the mediation analysis model (Model 4), with a sample size of 1000. The 95% confidence interval (CI) was used to determine the significance of the effect (significant if the CI does not include 0).

**Figure 9:**
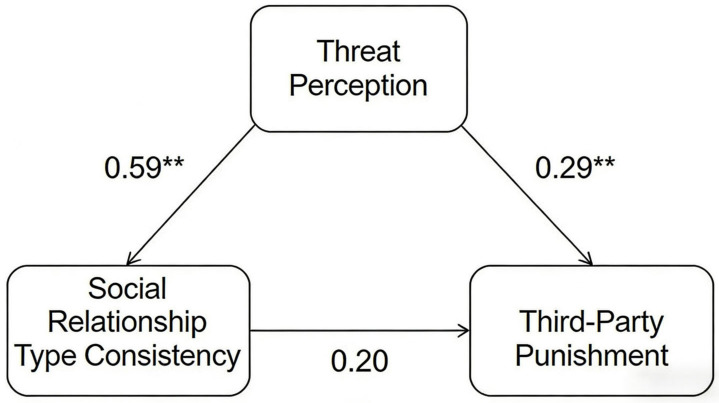
Mediation model with threat perception (slightly unfair scenario). Note. ** *p* < 0.0.

Using the level of punishment chosen by participants as the indicator for third-party punishment, the mediation analysis yielded the following results:

In the slightly unfair allocation scenario, the total effect of social relationship type congruence on third-party punishment was statistically significant (β = 1.94; *t* = 2.03; *p* < 0.05). This indicates that compared with incongruent social relationships, individuals engaged in more severe third-party punishment when social relationships were congruent. The mediating effect of threat perception between social relationship type congruence and third-party punishment was significant (β = 0.89; 95% CI = [0.23, 1.71]). This means that social relationship type congruence positively influenced threat perception, which in turn increased individuals’ third-party punishment. After controlling for the mediating effect of threat perception, the direct effect of social relationship type congruence on third-party punishment was not statistically significant (β = 1.05; *t* = 1.09; *p* = 0.28, 95% CI = [−0.86, 2.95]). This suggests that threat perception served as a full mediator, meaning that social relationship type congruence increased third-party punishment by elevating threat perception. The specific path coefficients are shown in Figure 9.

**Figure 10:**
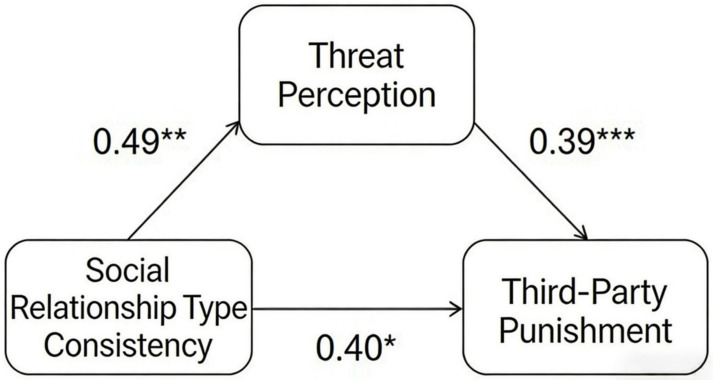
Mediation model of threat perception (extremely unfair scenario). Note. * *p* < 0.05, ** *p* < 0.0, *** *p* < 0.001.

In the extremely unfair allocation scenario, the total effect of social relationship type congruence on third-party punishment was statistically significant (β = 8.26; *t* = 3.26; *p* < 0.01). The mediating effect of threat perception was also significant (β = 2.72; 95% CI = [0.77, 4.73]), indicating that social relationship type congruence increased third-party punishment by positively influencing threat perception. After controlling for this mediation, the direct effect of social relationship type congruence on third-party punishment remained statistically significant (β = 5.54; *t* = 2.30; *p* < 0.05, 95% CI = [0.77, 10.31]). This indicates that threat perception served as a partial mediator. Social relationship type congruence not only increased third-party punishment by raising the threat perception but also directly promoted it. The specific path coefficients are shown in Figure 10.

#### 3.4.6. Discussion of Experiment 3

This experiment investigated the impact of social relationship type congruence on third-party punishment and the mediating role of threat perception. The results support our experimental hypotheses. The mediation analysis indicated that in the slightly unfair scenario, threat perception played a full mediating role between social relationship type congruence and third-party punishment. Specifically, congruent social relationships elicited higher threat perceptions, leading individuals to engage in more third-party punishment, whereas incongruent social relationships elicited lower threat perceptions, resulting in less punishment.

In the extremely unfair scenario, threat perception played a partial mediating role. Specifically, congruent social relationships led individuals to engage in more third-party punishment, while incongruent relationships led to less punishment. Furthermore, congruent social relationships elicited higher threat perceptions, which increased punishment, whereas incongruent relationships elicited lower threat perceptions, decreasing punishment. Additional ANOVA analyses, conducted separately for congruent and incongruent conditions, yielded results that were consistent with Experiment 2, demonstrating the stability of the experimental design and manipulations.

## 4. Discussion and Conclusions

### 4.1. General Discussion

Based on the influence of social relationships on third-party punishment, the present study further investigated the effect of relationship type congruence on third-party punishment and the mediating role of threat perception. Four experiments were conducted, and the results revealed that consistency in social relationship types affected individuals’ third-party punishment, with threat perception playing a mediating role in this relationship. The findings of this study go beyond the intuitive conclusion that “individuals are more lenient toward friends” and provide three progressive new insights for theories of third-party punishment. First, the critical role of “relationship type congruence” is proposed. The present study found that what determines punishment intensity is not mere social distance, but the consistency of the transgressor’s behavior within the relational network. This accounts for seemingly contradictory findings in previous research (e.g., why individuals sometimes punish friends more severely) and offers a more generalizable predictive variable. Second, the psychological mechanism of deterrent motivation is revealed. By verifying the mediating role of threat perception (Hypothesis H4), this study translates deterrence theory from abstract assumptions into a concrete cognitive pathway. The findings indicate that punishment serves not only as a reactive sanction against norm violations but also as a proactive defense based on risk assessment. Third, different theoretical perspectives are integrated. This study builds a bridge between social identity theory (emphasizing group identity) and deterrence theory (emphasizing self-interest). The results demonstrate that group identity (ingroup vs. outgroup) ultimately drives punishment by influencing individuals’ risk calculations within the relational network, thereby providing a more unified and explanatory theoretical model

#### 4.1.1. Self-Interest and Altruism: Explaining TPP Through Deterrence Theory

Based on deterrence theory, third-party punishment can be understood as an adaptive behavior that is essentially “self-interested.” The theory posits that an important motivation for individuals to enact punishment is to prevent themselves from experiencing unfair treatment in the future, reflecting the safeguarding of long-term self-interest (Krasnow et al., 2016). In this study, when the social relationship type between the violator and bystander is congruent with that between the violator and victim, the bystander more readily projects the victim’s situation onto themselves, thereby perceiving a higher level of personal threat. This heightened threat perception activates the individual’s self-protective motivation, prompting them to administer more severe third-party punishment to deter the violator and reduce their own future risk. This finding directly supports the core proposition of deterrence theory: the underlying driver of third-party punishment stems from the strategic protection of one’s own interests.

From the perspective of signal transmission, the bystander’s punitive behavior conveys two signals to the violator simultaneously: first, a deterrence signal, indicating that violating norms will incur costs and thereby discouraging future transgressions; second, a normative signal, reaffirming the existence of fairness norms within the group and encouraging behavioral adjustment (Guo et al., 2024). The consistency effect observed in this experiment precisely strengthens the “targeted” nature of this signal transmission: when the bystander and victim occupy the same relational position, the signal conveyed by the punishment—“if you treat him this way, you will treat me the same way”—becomes clearer and more forceful, thereby enhancing the credibility and normative weight of the deterrence.

Although deterrence theory emphasizes self-interested motivation, third-party punishment functionally achieves a synergy between individual and social interests. Punishment yields significant prosocial benefits by upholding social norms, protecting victims, and promoting group cooperation (Henrich et al., 2006). However, from the logic of deterrence, this prosocial benefit can be viewed as a “by-product” of self-interested motives: by maintaining group norms, individuals ultimately create a fairer, more predictable, and safer environment for themselves (Rand et al., 2012). Therefore, while third-party punishment may be motivated by self-interest, its outcome achieves a unification of prosocial and self-interested goals—a crucial foundation for its evolutionary persistence and its role in sustaining large-scale social cooperation.

#### 4.1.2. Punitive Deterrence and Normative Signaling: Viewing TPP from a Signaling Perspective

From an individual perspective, the need for self-protection serves as a key motivation driving third-party punishment and the release of deterrent signals. Threat perception plays a critical role in this process: when an individual observes that the social relationship type between the violator and victim is congruent with that between the violator and themselves, they are more likely to project the victim’s experience onto themselves, thereby feeling a stronger sense of threat (Delton & Krasnow, 2017). This heightened threat perception activates self-protective motives, prompting the individual to deter the violator through third-party punishment and thereby reducing the likelihood of experiencing unfair treatment in the future (Carlsmith et al., 2002). The findings of this study align with existing research; for example, when individuals perceive a higher level of threat, they tend to engage in more proactive self-protective behaviors, including aggressive responses (Zou et al., 2025). Krasnow et al. (2016) also found that when bystanders anticipate that they might receive the worst treatment from the violator, the intensity of their punishment increases significantly. In this study, third-party punishment can be viewed as an active self-protection strategy that conveys deterrent signals by sanctioning those who violate fairness norms (Guo et al., 2024), thereby reducing the individual’s future risk of encountering unfairness. Thus, threat perception not only explains the motivational basis of third-party punishment but also reveals how consistency in social relationship types regulates behavioral responses by influencing an individual’s psychological state (e.g., threat perception).

From a group perspective, maintaining good cooperation within a group relies on third-party punishment to transmit normative warning signals. The impact of social relationship type consistency on third-party punishment reflects the dynamic process of upholding fairness norms within groups. In a group setting, social relationships are a key factor shaping individual behavior. When the social relationship type between the violator and bystander is congruent with that between the violator and victim, the bystander is more likely to perceive the victim’s situation as a potential threat to themselves (Krasnow et al., 2016). This consistency enhances the bystander’s sensitivity to intra-group norms, motivating them to uphold these norms through third-party punishment (Kahan, 1996). S. Chen et al. (2021) refer to the phenomenon whereby third-party punishment activates social norms and promotes a cooperative group atmosphere as the “spillover effect of punishment.” Furthermore, punishment within a group serves not only as a sanction against the violator but also as a social normative signal (H. Chen et al., 2020), aiming to warn other members against violating group fairness standards. By punishing the violator, the bystander conveys a clear message to the group: violations of norms will be punished, thereby encouraging both the violator and other members to adhere to social norms (Peters et al., 2017; Wenzel & Thielmann, 2006). Such signals help strengthen normative identification within the group and promote the formation of cooperative behaviors. Thus, consistency in social relationship types indirectly fosters the maintenance of group norms and the consolidation of cooperative mechanisms by influencing the bystander’s threat perception.

From a societal perspective, the evolution of social fairness norms relies on the continuous implementation of third-party punishment. The role of threat perception in third-party punishment reflects the psychological foundation of social norm evolution (Henrich et al., 2006). Compared with second-party punishment, which is often driven by emotions, third-party punishment is more objective and neutral, focusing more on the norms themselves rather than personal emotions (Jordan et al., 2016). The persistence and evolution of social norms depend, in part, on individuals’ willingness to punish norm-violating behavior (Fehr & Gächter, 2000). When individuals perceive a higher level of threat, their willingness to punish increases, leading them to participate more actively in upholding social norms. This threat perception-based punitive behavior not only helps suppress violations but also provides intrinsic motivation for the dynamic evolution of social norms. In this study, consistency in social relationship types enhanced bystanders’ willingness to engage in third-party punishment by elevating their threat perception. This mechanism holds significant implications at the societal level: first, it explains why individuals are more likely to intervene in norm maintenance under specific social relationship conditions; second, it reveals the potential function of threat perception in the formation and reinforcement of social norms—when members of society generally perceive a higher level of threat, the enforcement of social norms may intensify, thereby promoting overall social stability and cooperation.

### 4.2. Limitations and Future Directions

Building on previous research and grounded in deterrence theory, this study explored the effect of social relationship type congruence on TPP in unfair events and its underlying psychological mechanism, enriching prior work. However, this study has several limitations that future research could address.

First, the manipulation of social relationships in this study was limited to two levels: friends and strangers. Real-life social relationships are far more complex. Future research could expand on these categories to improve ecological validity.

Second, the experimental design in this study was primarily based on scenario imagination tasks, which may not fully capture individuals’ real-life choices. Future studies could enhance situational realism through improved experimental designs to investigate more authentic reactions.

Third, the present study concentrated on the core mechanism and did not include diverse internal psychological factors and external social factors that may also influence punitive decision-making. This represents a boundary of the current research framework. Future research may integrate broader individual and social contextual variables into the model to examine their moderating roles. Such attempts will help identify the boundary conditions of the observed effects and enhance the generalizability and explanatory depth of the findings in real-life social situations.

Fourth, although the present study targeted adults, only college students were included as participants. Given the relatively homogeneous nature of this sample, the generalizability of the findings to the broader adult population is limited. Caution is needed when extending the current results to other adult groups. Future research should recruit more diverse adult samples across different ages, occupations, and social backgrounds to improve the generalizability of the findings.

Finally, this study explored the psychological mechanism of how social relationship type congruence influences TPP at the behavioral level but did not investigate the underlying neural mechanisms.

## Data Availability

The datasets generated during and/or analyzed during the current study are available from the corresponding author upon reasonable request.

## Supplementary Materials

The following supporting information can be downloaded at: https://www.mdpi.com/article/10.3390/bs16040482/s1, Table S1. Demographic Characteristics of Participants Across Four Experiments. Table S2. Shapiro-Wilk Normality Test Results for Dependent Variables. Table S3. Participant Exclusion Details Across Experiments. Text S1. Details of Outlier and Missing Data Handling. Text S2. Full Text of Scenario Manipulations.
