# Supplementary material for: The Influence of Social Relationships on Third-Party Punishment: The Roles of Relationship Type Congruence and Threat Perception
Source: Behav Sci (Basel). 2026 Mar 24;16(4):482. doi: 10.3390/bs16040482 (PMC13113965; doi:10.3390/bs16040482)
Supplement: Supplementary file 1 [file behavsci-16-00482-s001.zip › behavsci-4072058-supplementary.pdf]

Supplementary Materials

Table S1. Demographic Characteristics of Participants Across Four Experiments

Experiment	N	Gender (M/F)	Age M (SD)	Age Distribution (n)	Major (Arts/Science/Engineering)	Only Child (Yes/No)
Pilot	136	75 / 61	20.33 (1.10)	18 yr: 28; 19 yr: 36 20–21 yr: 53 ≥22 yr: 19	Arts: 51 Science: 38 Engineering: 47	Yes: 70 No: 66
Experiment 1	132	66 / 66	19.67 (1.51)	18 yr: 25 19 yr: 38 20–21 yr: 49 ≥22 yr: 20	Arts: 48 Science: 36 Engineering: 48	Yes: 67 No: 65
Experiment 2	120	69 / 51	23.06 (5.09)	18–20 yr: 28 21–23 yr: 42 24–26 yr: 35 ≥27 yr: 27	Arts: 45 Science: 39 Engineering: 48	Yes: 65 No: 67
Experiment 3	112	56 / 56	21.85 (2.35)	≤19 yr: 18 20–22 yr: 58 23–24 yr: 26; ≥25 yr: 10	Arts: 38 Science: 32 Engineering: 42	Yes: 57 No: 55

Note: Arts = psychology, sociology, Chinese language, etc.; Science = mathematics, physics, biology, etc.; Engineering = computer science, electronic information, mechanical engineering, etc.

Table S2. Shapiro-Wilk Normality Test Results for Dependent Variables

Experiment	Variable	W	p	Normality Assumption Met
Pilot	IOS score (relationship check)	0.968	.123	Yes
	Perceived fairness (fairness check)	0.972	.186	Yes
	Third-party punishment intensity	0.965	.098	Yes
Experiment 1	Perceived fairness (fairness check)	0.975	.231	Yes
	Third-party punishment intensity	0.969	.157	Yes

Experiment	Variable	W	p	Normality Assumption Met
Experiment 2	Similarity rating (congruence check)	0.969	.153	Yes
	Perceived fairness (fairness check)	0.974	.217	Yes
	Third-party punishment intensity	0.970	.189	Yes
Experiment 3	Similarity rating (congruence check)	0.972	.203	Yes
	Perceived fairness (fairness check)	0.968	.141	Yes
	Third-party punishment intensity	0.967	.128	Yes

Note: All dependent variables met the normality assumption ($p > .05$), justifying the use of parametric tests.

Table S3. Participant Exclusion Details Across Experiments

Experiment	Initial Recruitment	Exclusion Reason	Excluded (n)	Final Sample
Pilot	150	Failed manipulation check	10	136
		Outliers (3σ criterion)	4	
Experiment 1	150	Failed manipulation check	12	132
		Outliers / incomplete data	6	
Experiment 2	150	Failed manipulation check	20	120
		Outliers / incomplete data	10	
Experiment 3	150	Failed manipulation check	25	112
		Outliers / incomplete data	13	

Note: Exclusion criteria included incorrect responses to manipulation check questions, completion time <30 minutes, patterned responding, missing data >10%, and outlier values (>3 SD from mean).

Text S1. Details of Outlier and Missing Data Handling

Outlier detection. Outliers were identified using the 3σ criterion: values deviating more than three standard deviations from the group mean were excluded from analyses. Across all experiments, a total of 33 participants were excluded due to outliers (see Table S3 for per-experiment counts).

Missing data handling. Missing data rates were low across all experiments (<5%). For participants with missing data ≤10%, multiple imputation was performed using SPSS's MCMC algorithm with 5 imputations. Sensitivity analyses

comparing results with and without imputation showed consistent patterns, confirming that missing data did not bias the findings. Participants with missing data >10% were excluded entirely.

Text S2. Full Text of Scenario Manipulations

Friend Condition (Bystander–Transgressor = Friends)

"Imagine that A is someone you know very well, and you have a good relationship. Please identify a specific person in your mind and write down their name (nickname), age, and gender."

Stranger Condition (Bystander–Transgressor = Strangers)

No specific instruction; participants were informed that A is an unknown individual.

Transgressor–Victim Friend Condition (Experiment 1, Subgroup 1)

"Your friend A and his/her friend B have agreed to form a partnership to jointly complete a remunerative task. After the task, the reward will be uniformly issued to your friend A's account, and A will be responsible for distributing the reward between the two. As a bystander, you witness the entire task process and reward allocation."

Transgressor–Victim Stranger Condition (Experiment 1, Subgroup 2)

"Your friend A and a randomly selected individual B form a temporary partnership with no prior interaction or emotional bond. They jointly complete a remunerative task. After the task, the reward will be uniformly issued to your friend A's account, and A will be responsible for distributing the reward between the two. As a bystander, you witness the entire task process and reward allocation."

Congruent Condition: Both Friends (Experiment 2, Subgroup 1)

"Your friend A and his/her friend B have agreed to form a partnership to jointly complete a remunerative task. After the task, the reward will be uniformly issued to your friend A's account, and A will be responsible for distributing the reward between the two. As a bystander, you witness the entire task process and reward allocation."

Congruent Condition: Both Strangers (Experiment 2, Subgroup 2)

"A randomly selected individual A and a randomly selected individual B form a temporary partnership with no prior interaction or emotional bond. They jointly complete a remunerative task. After the task, the reward will be uniformly issued to A's account, and A will be responsible for distributing the reward between the two. As a bystander, you are a stranger to both A and B, and you witness the entire task process and reward allocation."

Incongruent Condition: Bystander–Transgressor Friends, Transgressor–Victim Strangers (Experiment 2, Subgroup 3)

"Your friend A and a randomly selected individual B form a temporary partnership with no prior interaction or emotional bond. They jointly complete a remunerative task. After the task, the reward will be uniformly issued to your friend A's account, and A will be responsible for distributing the reward between the two. As a bystander, you witness the entire task process and reward allocation."

Incongruent Condition: Bystander–Transgressor Strangers, Transgressor–Victim Friends (Experiment 2, Subgroup 4)

"A randomly selected individual A and his/her friend B have agreed to form a partnership to jointly complete a remunerative task. After the task, the reward will be uniformly issued to A's account, and A will be responsible for distributing the reward between the two. As a bystander, you are a stranger to A, and you witness the entire task process and reward allocation."
